# A MALDI-MS-based quantitative analytical method for endogenous estrone in human breast cancer cells

**DOI:** 10.1038/srep24489

**Published:** 2016-04-19

**Authors:** Kyoung-Jin Kim, Hee-Jin Kim, Han-Gyu Park, Cheol-Hwan Hwang, Changmin Sung, Kyoung-Soon Jang, Sung-Hee Park, Byung-Gee Kim, Yoo-Kyung Lee, Yung-Hun Yang, Jae Hyun Jeong, Yun-Gon Kim

**Affiliations:** 1 Department of Chemical Engineering, Soongsil University, Seoul 156-743, Korea; 2 School of Chemical and Biological Engineering, Seoul National University, Seoul 151-742, Korea; 3 Division of Bioconvergence Analysis, Korea Basic Science Institute, Chungbuk 363-883, Korea; 4 Division of Life Sciences, Korea Polar Research Institute, Incheon 406-840, Korea; 5 Department of Microbial Engineering, College of Engineering, Konkuk University, Seoul 143-701, Korea

## Abstract

The level of endogenous estrone, one of the three major naturally occurring estrogens, has a significant correlation with the incidence of post-menopausal breast cancer. However, it is challenging to quantitatively monitor it owing to its low abundance. Here, we develop a robust and highly sensitive mass-assisted laser desorption/ionization mass spectrometry (MALDI-MS)-based quantitative platform to identify the absolute quantities of endogenous estrones in a variety of clinical specimens. The one-step modification of endogenous estrone provided good linearity (R^2^ > 0.99) and significantly increased the sensitivity of the platform (limit of quantitation: 11 fmol). In addition, we could identify the absolute amount of endogenous estrones in cells of the breast cancer cell line MCF-7 (34 fmol/10^6^ cells) by using a deuterated estrone as an internal standard. Finally, by applying the MALDI-MS-based quantitative method to endogenous estrones, we successfully monitored changes in the metabolic expression level of estrones (17.7 fmol/10^6^ letrozole-treated cells) in MCF-7 cells resulting from treatment with an aromatase inhibitor. Taken together, these results suggest that this MALDI-MS-based quantitative approach may be a general method for the targeted metabolomics of ketone-containing metabolites, which can reflect clinical conditions and pathogenic mechanisms.

Estrogens are well known as the most important and ubiquitous steroid hormones in the female body and are responsible for sexual and reproductive development^1^. There are three major forms of estrogen (estrone (E1), estradiol (E2) and estriol (E3)) that occur naturally in women. Estrone (E1), which is predominantly found in postmenopausal women, is produced by the conversion of androstenedione *via* the enzyme aromatase^2^. As a growth hormone, the level of estradiol (E2) increases during pregnancy and may have an important role in the maintenance of pregnancy^3^. It is more potent than estrone owing to its unique chemical structure. Finally, estriol (E3) is only significantly generated from the placenta during pregnancy^4^. The biological effects of these endogenous estrogens are not restricted to effects on reproduction, as they travel through the bloodstream and play critical roles in a variety of physiological events. For example, they are involved in adipocyte development^5^, neuroendocrine and cerebral regulation^6^,^7^, immune cell function and cardiovascular function^6^,^8^,^9^.

The levels of circulating estrogens, including estrone (E1) and estrone sulfate, are positively related to the development and growth of breast cancer in postmenopausal females^10^,^11^,^12^,^13^. They participate in the proliferation and apoptosis of breast cells, increasing the likelihood of DNA mutations and carcinogenesis^14^. There have been several recent reports demonstrating the relationship between various estrogen metabolites and breast cancer risk^15^,^16^,^17^,^18^. The irreversible hydroxylation at the C-2, -4 or -16 positions of the steroid ring causes DNA damage that can increase the risk of breast cancer *via* various genotoxic pathways^15^,^17^,^18^. Consequently, estrone and estrone metabolites have been the main targets in studies of breast carcinogenesis and drug therapy mechanisms^10^,^11^,^12^,^13^,^14^,^15^,^16^,^17^,^18^. To monitor cancer development and the progress of the disease, however, highly sensitive and quantitative estrone analysis tools are required.

Conventional methods for the quantitative analysis of estrone or estrone metabolites include radioimmunoassay (RIA)^19^,^20^ and enzyme-linked immunosorbent assay (ELISA)^21^,^22^. However, these methods require an arduous antibody production for detection. Moreover, the concentrations of the circulating free estrones in the serum and plasma are occasionally lower than the limits of detection of the antibody-based assays (51.8 fmol in ELISA^21^, 74 fmol in RIA^20^). As a untargeted platform, gas chromatography- or liquid chromatography-tandem mass spectrometry (GC- or LC-MS/MS)-based analytical techniques have been used to physically separate highly complexed metabolites and accurately measure the circulating estrone concentration, which is correlated with breast cancer risk^23^,^24^,^25^,^26^,^27^,^28^. Tandem mass spectrometry-based methods can directly characterize the chemical structures of endogenous estrone metabolites with sufficient sensitivity, but these methods often are too complex, time-consuming and expensive for use in the clinical quantitation of low circulating estrone metabolites in postmenopausal females^23^,^24^,^26^,^29^,^30^,^31^,^32^,^33^. More recent studies have focused on the development of chemical derivatization methods (*e.g*., using dansyl chloride^24^,^26^, Girard’s reagent P (GP)^25^, or bis(trimethylsilyl) trifluoroacetamide (BSTFA)^28^) for steroid hormones to enhance the sensitivity of LC-MS/MS analysis. However, they require purification processes to remove excess chemical reagents, which can reduce the sensitivity of the electrospray-based detection.

To address these problems, matrix-assisted laser desorption/ionization mass spectrometry (MALDI-MS), which is an extremely sensitive and robust method that is suitable for the analysis of complex mixtures, could be a great alternative tool for the analysis of endogenous estrones^34^,^35^. Here, we demonstrate a MALDI-based quantitative platform for the quantitation of endogenous estrogenic hormones in human body fluid and cells (Fig. 1). First, we introduced a permanent cationic charge onto the ketone-containing E1 to simplify the MALDI spectra due to alkali metal adducts (*e.g*., Li^+^, Na^+^, or K^+^). The reliability of the quantitative method was validated using synthetic ketone-containing steroid hormones (*i.e.*, estrone and testosterone). After the chemical derivatization, there are significant improvements in the signal reproducibility and linearity (R^2^ > 0.99) compared to the underivatized counterparts. We used deuterated estrone (d4-E1) as an internal standard to monitor the change in the absolute amount of endogenous estrones in MCF-7 cells resulting from treatment with an aromatase inhibitor.

## Results and Discussion

### MALDI-MS-based quantitative analysis for ketone-containing synthetic estrones

**Figure 1:**
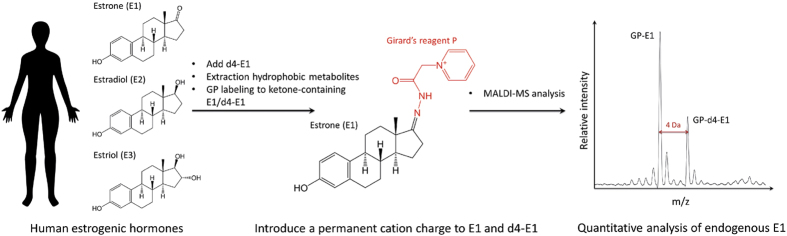
Schematic strategy of the MALDI-MS-based quantitative analysis of ketone-containing endogenous estrones.

Recently, our group has reported MALDI-MS-based quantitative analysis of carbohydrates and bioactive lipids (*e.g*., N-acyl homoserine lactones) that have an aldehyde or ketone group^34^,^35^,^36^. Previous studies have demonstrated that chemical derivatization of the target molecules could dramatically enhance the sensitivity (up to 6 × 10^4^ times) and allow more accurate quantitative analysis using only MALDI-MS without prior sample purification^36^. In this study, we expand the application to steroid hormones for the practical use of MALDI-MS in the quantitative analysis of targeted biomolecules. Figure 1 shows the workflow of the MALDI-MS-based quantitation of endogenous estrone. The total hydrophobic metabolites were collected *via* liquid-liquid extraction using MTBE, and the dried extracts were subsequently treated with Girard’s reagent P and subjected to MALDI-MS analysis without any extra purification steps. This robust method also enabled the measurement of the absolute quantity of endogenous estrones by adding deuterated estrone (*i.e*., d4-estrone) into human serum and MCF-7 cell lysate. To the best of our knowledge, this is the first report of a MALDI-MS-based method for quantifying endogenous estrogenic hormones.

**Figure 2:**
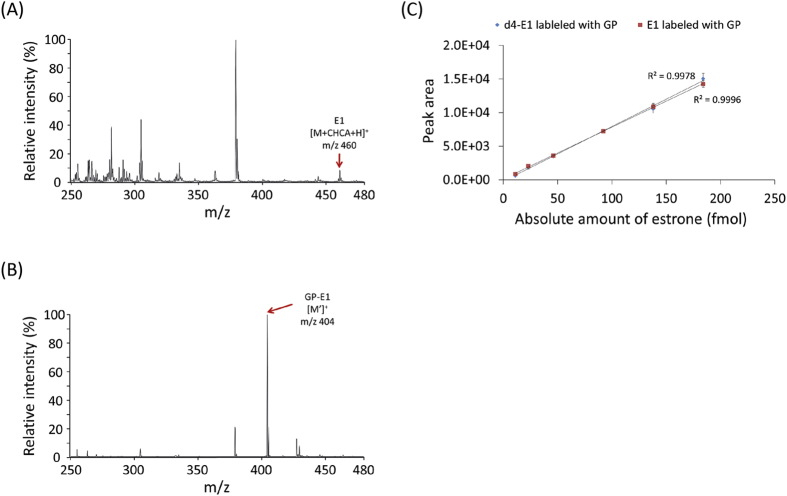
MALDI-MS spectra of (**A**) estrone (E1) and (**B**) GP-labeled estrone (GP-E1) with 185 pmol quantity on a MALDI spot and (**C**) a linear relationship between synthetic estrone quantity and peak area.

**Figure 3:**
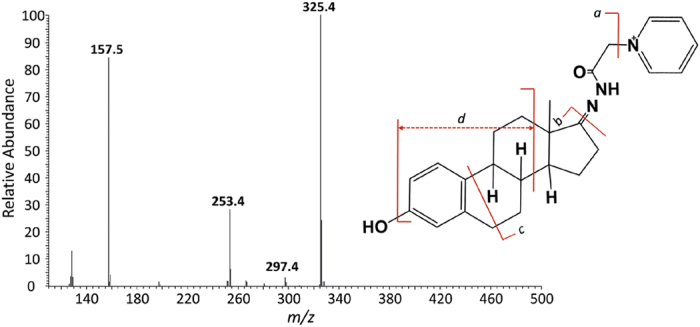
MS/MS profile of GP-labeled estrone: (**a**) *m/z* 325.4; (**b**) *m/z* 253.4; (**c**) *m/z* 297.4; (**d**) *m/z* 157.5.

To demonstrate the chemical derivatization method, synthetic estrone and testosterone, sex hormones possessing one ketone group, were used. First, the unlabeled estrone (E1) was identified at 460 *m/z* corresponding to the estrone-matrix adduct form (*i.e.*, [M + CHCA + H]^+^), as previously reported (Fig. 2A). The possible mechanism of matrix (*i.e.*, CHCA) adduct formation in MALDI spectra has been reported as π-π interactions or hydrogen bonds between the estrone and CHCA matrix^37^,^38^. However, chemical derivatization with Girard’s reagent P only enables the production of the [M′]^+^ ion for GP-E1 at 404 *m/z* without analyte-matrix adduct formation, which dramatically simplifies the MALDI profile (Fig. 2B). Moreover, liquid chromatography-tandem mass spectrometry (LC-MS/MS) analysis was carried out to validate the chemical conjugation of Girard’s reagent P onto the C17-ketone group of estrone. Figure 3 shows the positive ion MS/MS spectrum of the parent 404 *m/z* ion as GP-derivatized E1 ([M′]^+^). Among the MS/MS fragments, the appearance of a prominent **a** ion at 325 *m/z*, which contains the intact estrone and a partial GP fragment, confirms the coupling of Girard’s reagent P with an estrone molecule. More interestingly, the **c** ion at 297 *m/z* is structurally informative to demonstrate the position (*i.e*., C17-ketone of estrone) of the chemical derivatization, although it is relatively weak. The results indicate that the C17-ketone of estrone is highly reactive to the hydrazide moiety of Girard’s reagent P without steric hindrance. Likewise, the MALDI spectrum of synthetic testosterone shows both the protonated form at 289 *m/z* and the sodium adduct form at 311 *m/z*, while the two peaks were replaced with just one peak at *m/z* 422 after chemical derivatization with GP (Fig. S1). The GP-labeled estrone and testosterone showed improved signal resolution and signal-to-noise ratio (SNR) (up to 17 times) in the mass spectra compared to their unlabeled counterparts. Thus, our straightforward method enables a remarkable improvement in the quality of MALDI spectra for ketone-containing hormones and facilitates a more rapid and high-throughput analysis using a simple MALDI-MS instrument.

### Validation of the estrone detection method on estrone-spiked serum

To validate the quantitative linearity, accuracy and reproducibility of the MALDI-MS-based method, different concentrations of GP-E1 and GP-d4-E1 were examined. Figure 2C shows the quantitative correlation between the absolute amounts of the standards and the MALDI peak area. The standard calibration curves of both GP-estrone and GP-d4-estrone present good linearity (R^2^ > 0.99) in accordance with their absolute quantities. In addition, the MALDI peak intensities of GP-d4-E1 were precisely equivalent to those of GP-E1, which means that GP-d4-E1 is applicable for the absolute quantitation of GP-E1.

**Figure 4:**
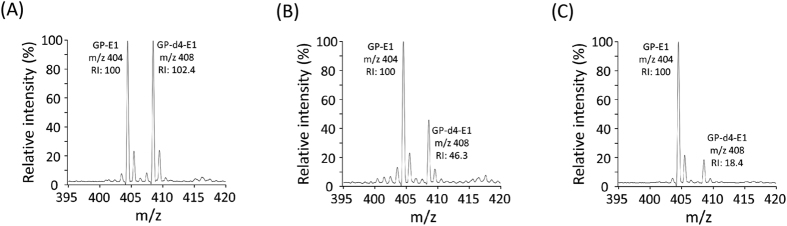
Relative quantitative analysis of estrone (E1) with different molar ratios of deuterated estrone (d4-E1) spiked in human normal sera. The molar ratios of E1 to d4-E1 were (**A**) 1:1, (**B**) 1:0.5, and (**C**) 1:0.2.

To verify that we can detect and quantify estrone and d4-estrone in complex media such as human serum and cell lysates, synthetic estrone and d4-estrone were spiked into human serum in various molar ratios (E1:d4-E1 = 1:1, 1:0.5, 1:0.2). After the MTBE extraction and subsequent GP labeling process, good quantitative recovery was observed without any purification process (Figs 4 and S2). Major peaks with a +4 Da difference in mass were clearly detected, as we intended. At the equivalent spiking (1:1) condition, the relative peak intensities were identical. In the more diluted ratios (1:0.5, 1:0.2), the peak intensities accurately reflected their theoretical molar ratios as well. In addition, we could determine the limit of quantitation (LOQ) of GP-estrone with the MALDI-MS-based quantitative method (Fig. S3). The GP derivatization significantly improved the LOQ of underivatized estrone (1.6 × 10^4^-fold, from 185 pmol to 11 fmol on the MALDI plate). Moreover, the GP-estrone spiked in human serum could be assayed at the femtomole level by MALDI-MS. According to the sensitivity of the MALDI-MS-based estrone quantitation, the endogenous estrones are sufficiently detectable. Taken together, these results demonstrate that the GP derivatization method remarkably improves the sensitivity and reproducibility of the endogenous estrones and provides reliable results in MALDI-MS-based estrone quantitation.

### Quantitation of endogenous estrone levels in MCF-7 cells

**Figure 5:**
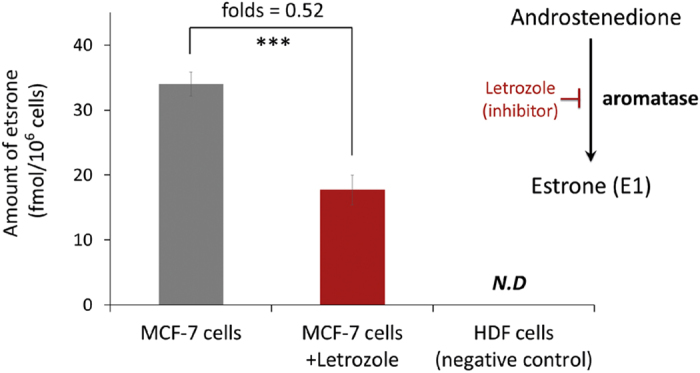
Bar graphs showing the decrease in endogenous estrone level in MCF-7 cells in response to the letrozole treatment. The intensities of GP-E1 from natural and letrozole-treated MCF-7 cells (each 10^6^ cells) correspond to 34 fmol and 17.7 fmol of estrone, respectively (***P value < 0.001; P values were derived from the two-tailed Student’s t test, n = 3; different MCF-7 cells/letrozole-treated MCF-7 cells. *Error bars* show SEM.).

Our MALDI-MS-based method was used to quantify endogenous estrone in cultured MCF-7 cells, a breast cancer cell line. After the lysis of the cells, d4-estrones were spiked for absolute quantitation of the endogenous estrones. The extracted estrones were labeled with GP and subsequently analyzed using our MALDI-MS-based method. Based on the peak intensity of d4-estrone (44 fmol), the amount of endogenous estrone was determined to be 34 fmol in 10^6^ MCF-7 cells (Figs 5, S4 and S5), which is comparable to a previous report (29 fmol estrone) and is physiologically realistic^39^,^40^. In addition, we would like to monitor changes in the levels of endogenous estrones. Letrozole has been effectively used as a medical therapy for hormonally responsive breast cancers because it can inhibit aromatase activity and thereby regulate endogenous estrone levels. The inhibitor was added to the cell culture, and then the cells were collected to quantify the endogenous estrone. As we expected, letrozole significantly decreased the absolute amount of estrone in the MCF-7 cells (Figs 5 and S5). An MTT assay was used to investigate the cytotoxic effects of letrozole on MCF-7 cells. As shown in Fig. S6A, no significant changes in the viability of cells were observed following treatment with less than 20 μM of letrozole. Treatment with 20 μM of letrozole for 48 h also does not affect the monolayered morphology of the cells (Fig. S6). Therefore, this treatment condition (*i.e.*, 20 μM of letrozole for 48 h) was used in following investigations. This result demonstrates that the MALDI-MS-based estrone quantitation method may be applicable for diagnosing and monitoring hormone-related diseases, such as breast cancers.

## Conclusion

In conclusion, we developed a highly sensitive and reliable MALDI-MS-based endogenous estrone quantitation method. To amplify the MALDI signals of estrone, Girard’s reagent P was used to label ketone-containing estrones to provide them with a permanent singly positive charge. Compared with previous approaches, the MALDI-MS-based estrone quantitation method offers several advantages. First, the method can rapidly and robustly quantify estrones from human serum or cell lysates without any need for additional chromatographic purification. Second, the use of deuterated estrone enables the quantification of the absolute amount of endogenous estrones. Third, the highly sensitive quantitation of estrones can be achieved at femtomole levels. Finally, the method can be applied to disease diagnosis or monitoring in the clinical setting. It is believed that the MALDI-MS-based quantitation method may contribute to targeted metabolite or hormone analysis of human specimens such as blood, urine, cells or tissues, which are directly related to various disease phenotypes.

## Materials and Methods

### Chemicals and Materials

Estrone, methyl tert-butyl ether (MTBE), α-cyano-4-hydroxycinnamic acid (CHCA), dimethyl sulfoxide (DMSO) and letrozole were purchased from Sigma-Aldrich (St. Louis, MO, USA). Methanol and acetic acid were obtained from Junsei Chemical (Tokyo, Japan). Girard’s reagent P (GP) was obtained from Tokyo Chemical Industry (Tokyo, Japan). Estrone-2,4,16,16-d4 was obtained from Cayman Chemical (Ann Arbor, MI). ReadyPrep proteomic-grade water was obtained from Bio-Rad Laboratories (Hercules, CA). Roswell Park Memorial Institute (RPMI-1640) medium, a phosphate-buffered saline (PBS) buffer, Hanks’ Balanced Salt solution (HBSS) and penicillin-streptomycin (Pen-Strep) were purchased from Gibco (Grand Island, NY). Fetal bovine serum (FBS) was obtained from the American Type Culture Collection (ATCC). Human normal sera were kindly provided by Prof. Jun Kyu Lee (Dongguk University Ilsan Hospital, Korea). The blood specimens are samples that were discarded under approved institutional review board protocols.

### Analysis of estrone and testosterone

First, 4 nmol of estrone and 0.04 nmol of testosterone in 99% methanol/1% acetic acid was prepared and reacted with Girard’s reagent P (GP) for 4 hr at room temperature (RT). Then, the mixture was analyzed using MALDI-TOF MS. The steps are described in detail below.

### Sample preparation and estrone extraction

Various concentrations of estrone (1, 10, 100 pmol, 1 nmol) were treated in 20 μL of human serum and subsequently mixed with 180 μL of PBS buffer. The extraction of estrone from the serum was performed as described previously. Briefly, 200 μL each of the serum sample and MCF-7 (10^6^ cells) lysate were mixed with 1.5 mL of MTBE for liquid-liquid extraction. After vortex-mixing for 10 min, the sample was incubated on ice for 20 min. The upper (organic) phase was collected and then dried under a stream of nitrogen gas. For the absolute quantification of endogenous estrones, 4.4 pmol (1.21 ng) of d4-estrone was added to the MCF-7 cell lysates prior to extraction.

### Girard’s reagent P (GP) derivatization

Dried extracts including estrone and/or d4-estrone were dissolved in 10 μL of 99% methanol/1% acetic acid. The solution was mixed with 100 μL of GP solution (5 mM in 99% methanol/1% acetic acid) and then incubated at RT for 4 hr. After incubation, the reactants were dried under vacuum without any additional clean-up steps.

### MALDI-MS analysis

The dried GP-conjugates were dissolved in 10 μL of 50% aqueous methanol. One microliter of the GP-estrone conjugates was mixed with 1 μL of CHCA matrix solution (10 mg/mL in 70% [v/v] acetonitrile/30% water). The mixture (1 μL) was spotted on a stainless steel MALDI plate, and the plate was dried at RT. Quantitative detection of estrone was performed on a Microflex LRF MALDI-TOF mass spectrometer in reflectron mode (Bruker Daltonics, Bremen, Germany). MALDI spectra were obtained by scanning a total of 1000 shots from 5 different spots in positive ion mode. The operating conditions were as follows: accelerating voltage = 20 kV, laser frequency = 60 Hz, ion source 1 voltage = 19 kV, ion source 2 voltage = 16 kV, lens voltage = 9.8 kV, detector gain = 5.8 and laser power = 25–35%. The intensity of each ion was obtained by summing from the first to the third isotopic peak areas. Spectral acquisition and processing were performed with FlexAnalysis software (ver. 3.3, Bruker Daltonics, Bremen, Germany).

### Cell assays

ER-positive human breast adenocarcinoma cell lines (MCF-7, Korean Cell Line Bank) with a passage number lower than three were used in this study. The MCF-7 cells were cultured in a Roswell Park Memorial Institute medium (RPMI-1640, Gibco) supplemented with 10% (v/v) fetal bovine serum (FBS, American Type Culture Collection, [ATCC]) and 1% (v/v) penicillin-streptomycin (P/S, Gibco). In parallel, human dermal fibroblasts (HDFs, ATCC) were cultured in a M106 medium supplemented with 2% (v/v) low serum growth supplement (Life Technologies) and 1% (v/v) P/S as a control cell line. The cells were cultured as monolayers at 37 °C in a 5% CO_2_ and 95% relative humidity environment. The medium was exchanged for a fresh medium every 3 days. The cells were treated for 48 hr with 20 μM of letrozole in FBS-free medium. The treated cells were collected using 0.25% trypsin and 0.04% EDTA in HBSS (Gibco) and lysed in 1× PBS prior to quantification of the estrone (E1) levels.

### MTT cell viability assay

The cytotoxic effects of letrozole on MCF-7 cells were quantitatively evaluated using an MTT cell proliferation assay kit (3-(4,5-dimethylthiazol-2-yl)-2,5-diphenyltetrazolium bromide, Invitrogen). The cells were treated with various concentrations of letrozole (up to 100 μM) for 24 hr and 48 hr. Solvent controls were conducted simultaneously using dimethyl sulfoxide (DMSO, Sigma Aldrich), with appropriate concentrations corresponding to each treatment. MTT reagent was added, and the cells were incubated for 4 hr. The absorbance of the sample altered with the positively stained metabolically active viable cells was subsequently measured at 570 nm using a microplate spectrophotometer (Multiskan Go., Thermo Scientific). The absorbance was normalized to that measured with untreated cells to quantify the relative cellular metabolic activity.

### LC-MS/MS analysis

To verify the conjugation of GP to estrone, tandem MS analysis was performed using an LCQ Deca XP ion trap mass spectrometer (Thermo Fisher Scientific, Waltham, MA) equipped with an Agilent 1100 HPLC system (Agilent Technologies, Santa Clara, CA). The analysis parameters were as follows: positive-ion mode, spray voltage of 5 kV, nitrogen sheath gas at a flow rate of 20 arbitrary units, and a heated capillary of 200 °C. 10 μL of the sample was injected and loaded into a Thermo 20 × 2.1 mm guard column at a flow rate of 8 μL/min in 50% ACN containing 0.1% formic acid. The tandem mass analysis of the GP-labeled estrone was performed manually, inducing a collision energy from 20 to 45 eV. The maximum ion collection time was set to 50 ms, and a 3 Da isolation width was used. All LC/MS data were processed by Xcalibur software (Thermo Electron, San Jose, CA).

## Additional Information

**How to cite this article**: Kim, K.-J. *et al.* A MALDI-MS-based quantitative analytical method for endogenous estrone in human breast cancer cells. *Sci. Rep.* **6**, 24489; doi: 10.1038/srep24489 (2016).

## Supplementary Material

Supplementary Information
