# Supplementary material for: A MALDI-MS-based quantitative analytical method for endogenous estrone in human breast cancer cells
Source: Sci Rep. 2016 Apr 19;6:24489. doi: 10.1038/srep24489 (PMC4836303; doi:10.1038/srep24489)
Supplement: Supplementary Information [file srep24489-s1.pdf]

Supplementary Information (SI)

A MALDI-MS-based quantitative analytical method for endogenous estrone in human breast cancer cells

Kyoung-Jin Kim[1], Hee-Jin Kim[1], Han-Kyu Park[1], Cheol-Hwan Hwang[1], Changmin Sung[2], Kyoung-Soon Jang[3], Sung-Hee Park[2], Byung-Gee Kim[2], Yoo-Kyung Lee[4], Yung-Hun Yang[5], Jae Hyun Jeong[1,*], Yun-Gon Kim[1,*]

[1]*Department of Chemical Engineering, Soongsil University, Seoul 156-743, Korea*

[2]*School of Chemical and Biological Engineering, Seoul National University, Seoul 151-742, Korea*

[3]*Division of Bioconvergence Analysis, Korea Basic Science Institute, Chungbuk 363-883, Korea*

[4]*Division of Life Sciences, Korea Polar Research Institute, Incheon 406-840, Korea*

[5]*Department of Microbial Engineering, College of Engineering, Konkuk University, Seoul 143-701, Korea*

(A)

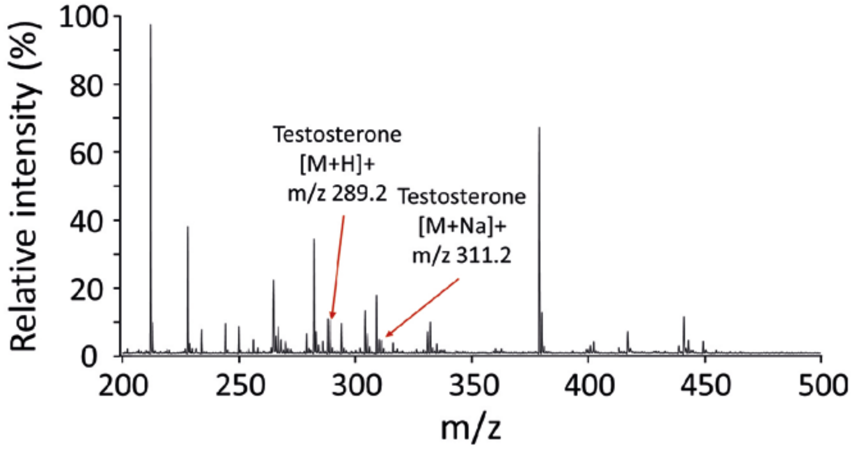

(B)

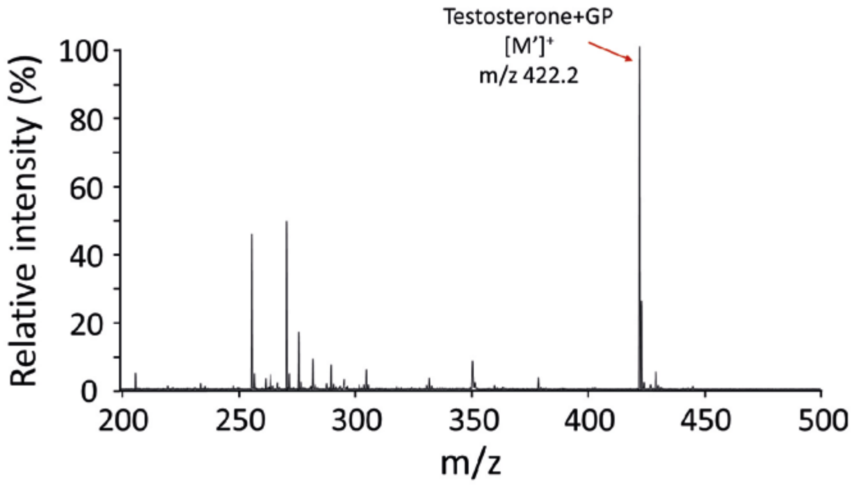

Supplementary Figure 1. MALDI-MS spectra of (A) testosterone and (B) GP-labeled testosterone (1.85 pmol on spot)

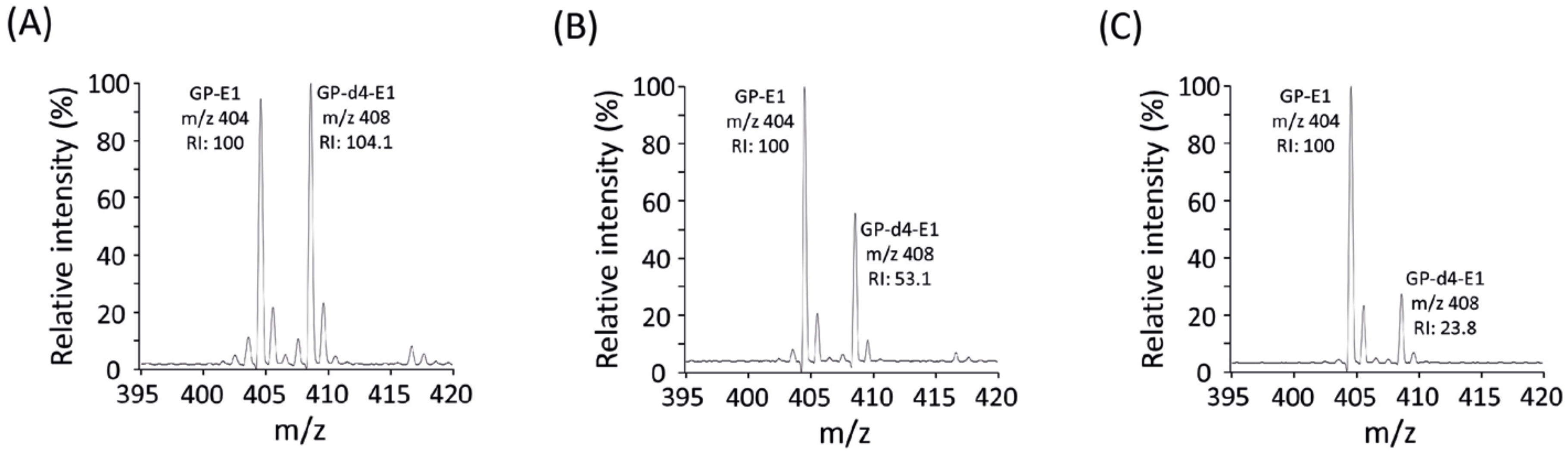

Supplementary Figure 2. Expanded MALDI spectra of various molar ratios of estrone:d4-estrone ((A) 1:1, (B) 1:0.5, (C) 1:0.2)

(A)

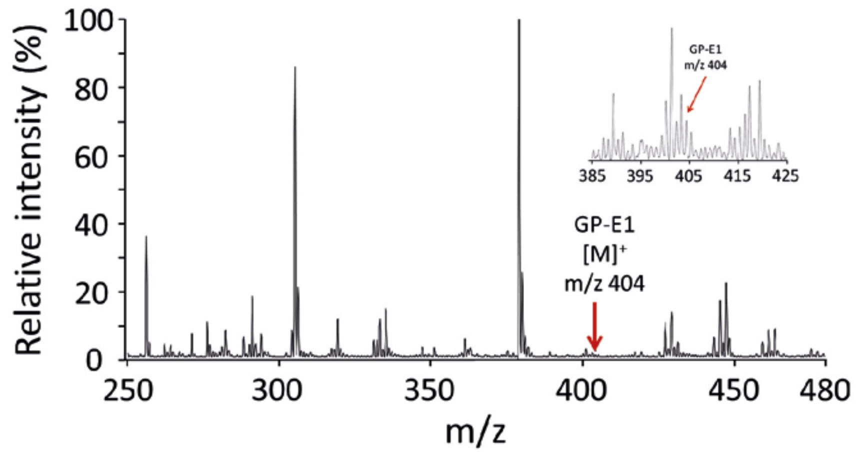

(B)

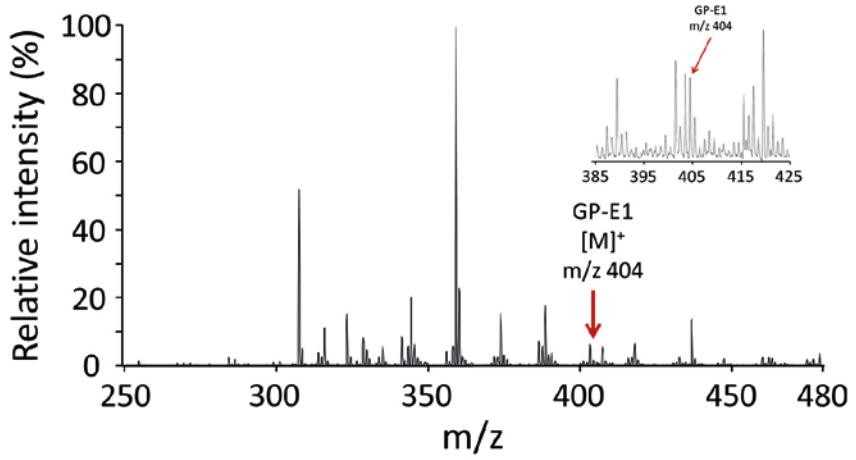

Supplementary Figure 3. MALDI-MS spectra of GP-labeled (A) estrone and (B) estrone spiked in human normal serum showing limit of detection (LOD)

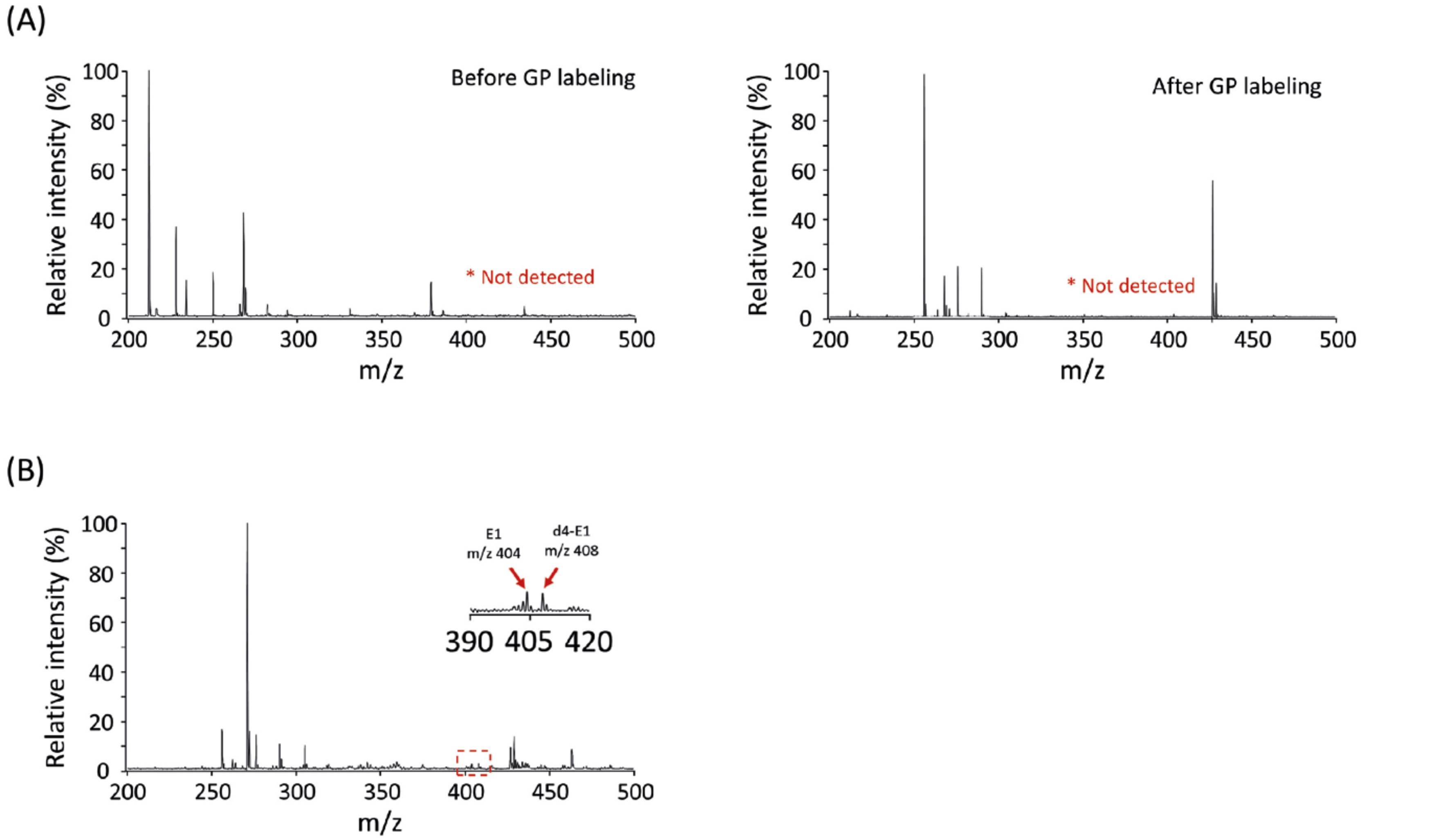

Supplementary Figure 4. MALDI spectrum of (A) E1 detected in HDF cells and (B) E1 from MCF-7 cells spiked in 8 nmol of d4-E1

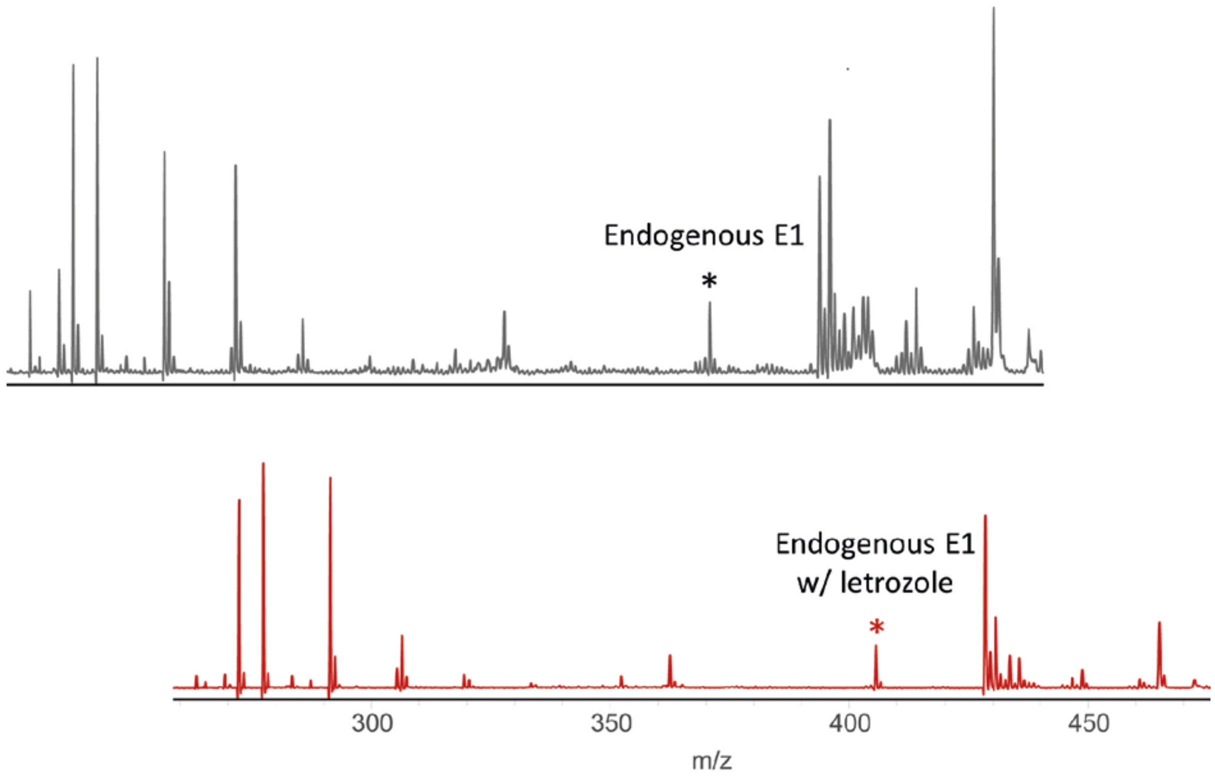

Supplementary Figure 5. MALDI-MS spectra of E1 detected in MCF-7 cells and in MCF-7 cells treated with 20 μM letrozole after GP derivatization

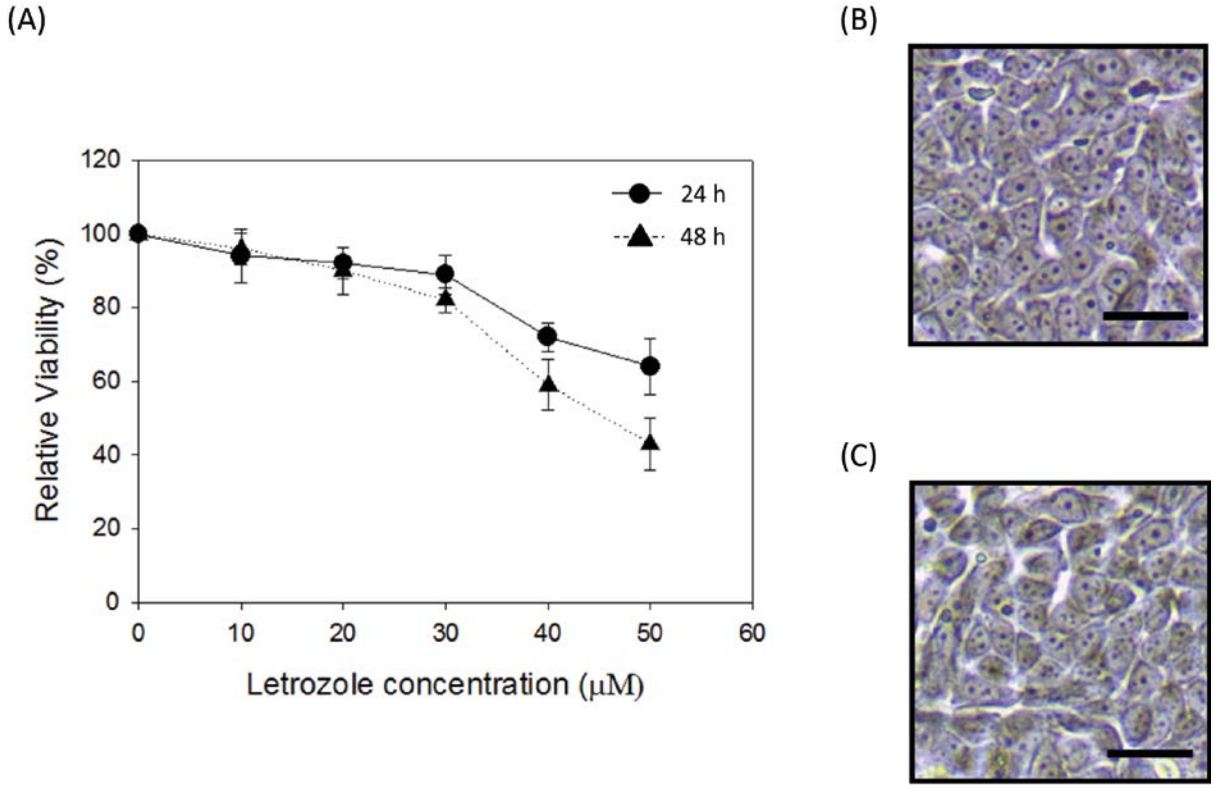

Supplementary Figure 6. (A) Cytotoxic effects of letrozole on MCF-7 cells over 24h (-●-) and 48h (-▲-). Letrozole below concentrations of 20 μM did not show serious effects on the viability of MCF-7 cells with 48h exposure. Solvent toxicity test was carried out with DMSO, and was found to induce insignificant levels of toxicity of that. All average data are presented as means ± SE. The cells treated with 20 μM letrozole for 48 h (C) were remained in monolayers like that of the untreated cells (B). Scale bars represent 50 μm.
